# Preliminary Feasibility Evaluation of Groove-Filling Thermochromic Polyurea Composites for Low-Temperature Visual Warning in Black Ice-Prone Pavements

**DOI:** 10.3390/polym18151860

**Published:** 2026-07-29

**Authors:** Junseo Lee, Hwang-Hee Kim, Yeeun Kim, Chul-Hyun Won, Gyeongho Lee, Kwan Kyu Kim, Jaeheum Yeon

**Affiliations:** 1 Interdisciplinary Program in Earth Environmental System Science & Engineering, Kangwon National University, Chuncheon 24341, Republic of Korea; ejun0208@kangwon.ac.kr; 2 GK Tech Co., Ltd., Chuncheon 24210, Republic of Korea; hwang1032@gktech.co.kr (H.-H.K.); yeeun@gktech.co.kr (Y.K.); 3 Korea Conformity Laboratories, Chuncheon 24341, Republic of Korea; okaoka@kcl.re.kr; 4 Gangwon State Office Relocation Task Force, Chuncheon 24266, Republic of Korea; rudhdi8585@korea.kr; 5 Department of Regional Infrastructure Engineering, Kangwon National University, Chuncheon 24341, Republic of Korea

**Keywords:** black ice-prone pavement, thermochromic pavement material, reversible thermochromic pigment, transition temperature, CIE L*a*b* colorimetry, CIE 1931 chromaticity, groove-filling polyurea composite

## Abstract

Black ice poses a serious winter road-safety hazard because its transparent appearance and localized formation make dangerous pavement conditions difficult to detect before vehicles enter affected areas. Converting this hidden low-temperature risk into visible surface information is therefore important for intuitive driver warning and road management. In this study, groove-filling thermochromic polyurea-based composites were fabricated using aliphatic polyurea resin, reversible thermochromic pigment (R.T.P.), and silica sand, and their colorimetric response, mechanical properties, and groove-filling applicability were investigated. Considering visual response and mechanical performance, the mixture containing 10 wt.% binder and 20 phr R.T.P. was selected as the most balanced candidate. For this mixture, the CIE L*a*b* values changed from 58.36, 3.85, and 13.32 for the control specimen to 47.82, 22.95, and 8.52, respectively, indicating a darker and more reddish appearance. The maximum compressive and flexural strengths were 10.13 MPa and 6.20 MPa, respectively. In addition, the selected mixture retained its low-temperature color response in the groove-filled state, confirming its preliminary groove-filling applicability. These results suggest the potential of thermochromic polyurea-based composites as visual-warning pavement materials for winter road safety.

## 1. Introduction

In winter road environments, pavement friction and driving conditions can change rapidly due to low air and pavement surface temperatures, snowfall, rainfall-induced refreezing, and variations in solar radiation [1,2,3]. These environmental changes can alter tire–pavement contact and vehicle stability, thereby compromising winter road safety [4,5]. Accordingly, various material- and maintenance-based approaches have been investigated to mitigate winter pavement hazards, including snow-melting asphalt materials, microwave-assisted deicing technologies, and studies on tire–pavement friction under thermo-mechanical conditions [1,4,5,6]. Among these hazards, black ice is particularly critical because it can directly threaten road operation and traffic safety while remaining difficult to recognize in advance [7,8].

Black ice forms as a thin and transparent ice film on the pavement surface and often appears similar to a wet road surface, making visual identification more difficult than for ordinary snow-covered or visibly icy roads [9,10,11,12]. Because its formation is governed by the combined effects of air temperature, pavement surface temperature, humidity, precipitation, solar radiation, and local surface conditions, black ice tends to occur locally and irregularly rather than uniformly over an entire road section [7,8,9]. Once formed, the frozen or low-friction surface can reduce tire–pavement friction and anti-skid performance, increase braking distance, and cause skidding, steering instability, rear-end collisions, and vehicle rollover [10,11,12,13,14,15,16]. Therefore, black ice is not only a pavement-surface condition problem but also a road-safety issue that requires early recognition and effective warning.

Various approaches have been developed to manage black ice risk, including meteorological monitoring, pavement-temperature measurement, image-based analysis, IoT- and sensor-based road-icing detection, AI-based recognition, UAV-based observation, patrols of ice-prone road sections, and snow-removal or deicing operations [17,18,19,20,21,22,23,24]. These approaches are useful for detecting or predicting icy pavement conditions and supporting maintenance decisions; however, they generally rely on external sensing devices, fixed observation points, data-processing systems, patrol-based inspection, or follow-up maintenance operations.

Because black ice is often localized, visually subtle, and irregularly distributed, detection- or device-based warning approaches may not always provide immediate and intuitive information at the exact pavement location where the hazardous condition occurs [25,26,27]. This limitation highlights the need for a responsive pavement material that can directly indicate low-temperature hazardous conditions on the pavement surface and help road users recognize risky sections in advance for timely speed reduction or route adjustment.

Stimulus-responsive materials have therefore attracted attention as a potential material-based approach for road safety applications [28,29]. In particular, reversible thermochromic pigment (R.T.P.) is a functional material that changes color in response to temperature variation and returns to the original color when the temperature recovers, without requiring an external power supply or electrical sensor [30,31,32]. R.T.P. is generally produced as microcapsules containing leuco dyes, developers, and solvents that induce coloration or decolorization within a designed temperature range [33,34]. The microcapsule structure protects the thermochromic components from the external environment and helps maintain reversible color-changing functionality [35,36]. Because the thermochromic transition temperature can be controlled through material design, R.T.P. can provide a threshold-temperature response for visually indicating low-temperature pavement conditions associated with icing risk [37,38].

Thermochromic response should provide not only color change but also sufficient color contrast, lightness variation, and visibility for drivers or road managers to intuitively recognize low-temperature hazardous conditions [39,40]. These color-development characteristics are influenced not only by the transition temperature and dosage of the thermochromic pigment, but also by the optical transparency, pigment dispersibility, refractive characteristics, and cured surface condition of the binder system [41]. Therefore, stable implementation of thermochromic functionality on road surfaces requires an appropriate polymer matrix that can fix and protect the pigment while preserving its reversible color-changing behavior [42].

Considering these material requirements, recent studies on thermochromic and temperature-responsive materials provide guidance for developing pavement materials that convert temperature changes into visible information. Zhan et al. [43] developed a cold-responsive visual indicator based on a gold–liquid crystal elastomer bilayer structure. A decrease in temperature induced deformation of the liquid crystal elastomer and fracture of the gold layer, leading to the generation of an optical signal. This demonstrates that a critical low-temperature condition can be translated into a measurable visual response through material design, and the resulting color signal can be quantitatively evaluated using CIE 1931 chromaticity coordinates [44].

In pavement materials, thermochromic pigments have mainly been incorporated into asphalt binders or coatings to modify optical and thermal behavior. Hu et al. [45] developed thermochromic asphalt binders containing red, blue, and black thermochromic powders and evaluated both spectral reflectance and Superpave-based binder properties. The study showed that the incorporation and dosage of thermochromic powders affected not only reflectance but also penetration, softening point, viscosity, complex modulus, rutting parameter, fatigue parameter, and stiffness. This indicates that thermochromic pigment addition should be considered as a mixture-design variable that can influence both visual/optical response and mechanical or rheological performance. Hu and Yu [46] further applied thermochromic materials as asphalt coatings and characterized thermal, optical, and chemical properties, demonstrating that the binder or coating matrix can affect how thermochromic pigments express their optical response on pavement surfaces.

The durability and stability of thermochromic functionality are also critical for pavement applications. Chen et al. [47] investigated the long-term photo-oxidation aging behavior of thermochromic bitumen containing reversible thermochromic microcapsules. Combining accelerated UV aging, rheological characterization, chemical structure analysis, and micromorphological observation, their study showed that the long-term performance of thermochromic pavement materials depends not only on the initial color-changing function but also on the stability of microcapsules and their interaction with the surrounding matrix. This is crucial for polymer-based thermochromic composites because the pigment shell, binder environment, and filler structure can affect pigment protection, dispersion, and long-term color response.

More recent pavement-coating studies have emphasized that thermochromic functionality should be evaluated together with colorimetric response, pigment composition, and road-service performance. Zhang et al. [48] developed red and black thermochromic pavement coatings and evaluated spectral reflectance, CIE L*a*b* color parameters, and thermal behavior. Their results suggest that surface-exposed thermochromic systems allow the optical response of pigments to be more directly observed than binder-embedded systems. Li et al. [49] developed a composite coating combining a thermochromic layer with a snow-melting and ice-suppression layer, and evaluated light aging, ice-melting performance, skid resistance, and road performance in addition to reflectance and thermal behavior. This study shows that recent thermochromic pavement materials are increasingly assessed not only by color or thermal response but also by performance indicators required for road application. Chen et al. [50] developed a red thermochromic composite coating and optimized the ratio between thermochromic red pigment and inorganic red pigment, indicating that pigment composition can strongly affect colorimetric and optical performance. These studies collectively highlight the need to evaluate thermochromic pavement materials from a mixture-design perspective rather than treating the pigment response alone as sufficient.

For direct visual warning under winter road conditions, Lima Jr. et al. [51] developed thermochromic road markings by incorporating thermochromic microcapsules with a transition temperature near 0 °C into acrylic road-marking paint. The study evaluated CIE L*a*b* color parameters, color difference ΔE and retroreflected luminance over a temperature range from −15 to 25 °C. The road markings changed from white to a pinkish hue below 0 °C, and the color difference reached up to 43, demonstrating that thermochromic materials can provide a strong visual signal for ice- and snow-related warning. However, this approach was based on a road-marking paint system and did not address the development of a load-resisting polymer composite or its applicability as a localized pavement filling material. These studies are summarized in Table 1.

Overall, previous studies demonstrate that thermochromic materials can generate temperature-dependent color or optical responses in pavement-related systems. However, most studies have focused on asphalt binders for thermal regulation, surface coatings for pavement cooling, or road markings for visual warning. These approaches provide useful insights into thermochromic functionality, but they do not fully address the development of a localized pavement-filling composite that can combine visible low-temperature response with basic mechanical integrity.

In particular, limited research has been conducted on thermochromic polymer composites designed as localized groove-filling pavement materials that simultaneously consider low-temperature color response, pigment–binder–filler mixture design, and basic mechanical performance. Therefore, this study investigates the effects of mixture proportion on the colorimetric response and basic mechanical performance of thermochromic polyurea-based composites and evaluates their preliminary applicability as a groove-filling material for black ice-prone pavement sections. The main objectives of this study are as follows:

- To establish mixture proportions of thermochromic polymer composites by varying binder content and R.T.P. dosage.
- To quantitatively evaluate the low-temperature color response and basic mechanical performance of the composite mixtures.
- To select a balanced candidate mixture and assess its preliminary groove-filling applicability using a groove-simulated concrete panel.

## 2. Materials and Methods

### 2.1. Materials

In this study, a polymer binder, reversible thermochromic pigment (R.T.P.), and an inorganic filler were used as the main constituent materials to prepare temperature-responsive polymer composites capable of visually indicating low-temperature pavement conditions associated with black ice risk. The polymer binder was used as the matrix material to provide bonding capacity, curing stability, and applicability to pavement surfaces. The R.T.P. was incorporated as the functional component responsible for color development under low-temperature conditions. Silica sand was used as an inorganic filler to improve volumetric stability, workability, and surface durability of the composite.

**Figure 1:**
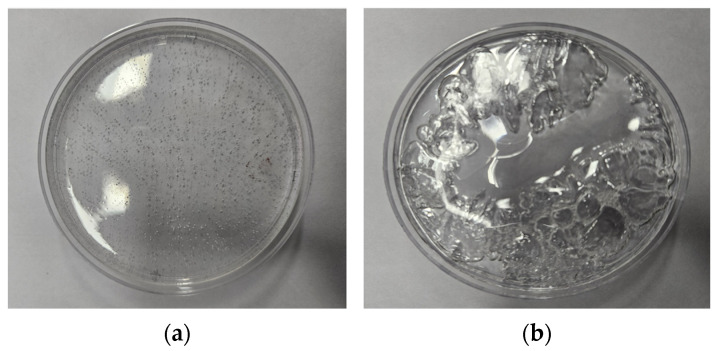
Comparison of candidate resins for thermochromic polymer composites: (**a**) aliphatic polyurea resin and (**b**) bio-based polyurethane resin.

Aliphatic polyurea resin and bio-based polyurethane resin were initially considered as candidate polymer binders. Both resins were selected as transparent binder candidates because optical transparency is important for preserving the color development of thermochromic pigments. In the preliminary evaluation, the bio-based polyurethane resin showed acceptable transparency; however, it required a longer curing time of approximately 90 min, and bubble formation was observed during curing. In contrast, the aliphatic polyurea resin showed good transparency without noticeable bubble formation during curing and exhibited a relatively short curing time of approximately 20 min in the preliminary curing test. Therefore, the aliphatic polyurea resin was selected as the final binder in this study, considering color visibility, curing stability, and preliminary applicability as a pavement filling material. The appearance of the candidate resins is shown in Figure 1, and the basic properties of the aliphatic polyurea resin used in this study are summarized in Table 2.

The aliphatic polyurea resin used in this study is a two-component resin that cures through the reaction between an amine-containing component and an isocyanate-containing component. During curing, amine groups react with isocyanate groups to form urea linkages, resulting in the formation of a cured polyurea network. This cured network serves as the polymer matrix that fixes the R.T.P. and silica sand within the composite.

The reversible thermochromic pigment (R.T.P.) used in this study was manufactured by InSilico Co., Ltd., Ansan, Republic of Korea, and was used as the functional material for temperature-dependent color development [52]. This pigment changes color in response to temperature variation and returns to its original color when the temperature recovers, indicating reversible thermochromic behavior. The pigment has a microcapsule-based structure, in which the outer shell protects the internal thermochromic components, while the active material mixture in the core induces color transition according to temperature change. In this study, the R.T.P. was applied as the main functional pigment for visually indicating low-temperature pavement conditions. The basic properties of this pigment are summarized in Table 3.

Silica Sand No. 5, supplied by Kyungin Materials Co., Ltd., Gimpo, Republic of Korea, was used as the inorganic filler. The particle size of the silica sand was 1.25 mm. The silica sand was incorporated to fill pavement grooves together with the polymer binder and to enhance the volumetric stability and surface durability of the cured composite. The basic properties of the silica sand are summarized in Table 4.

**Figure 2:**
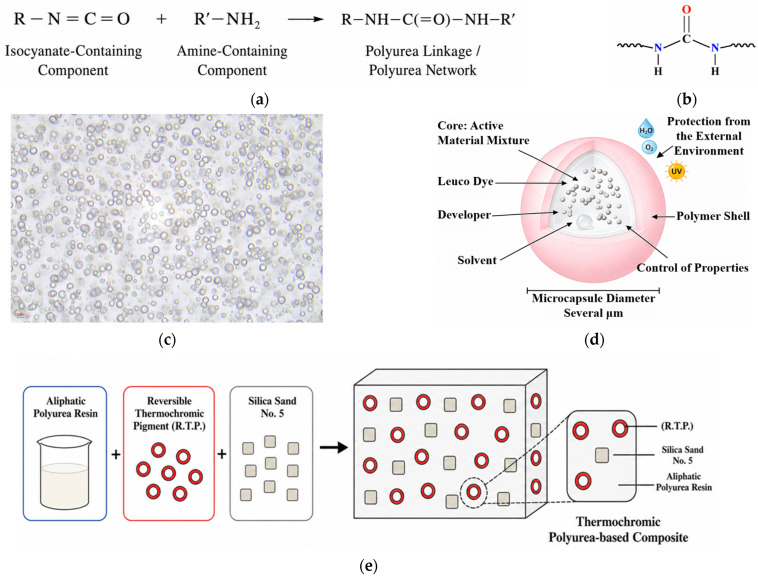
Schematic overview of the constituent materials and composite system: (**a**) polyurea curing reaction, (**b**) urea linkage structure, (**c**) microscopic image of R.T.P. microcapsules, (**d**) core–shell structure of R.T.P. microcapsules, and (**e**) thermochromic polyurea-based composite.

The representative curing reaction, R.T.P. morphology, and composite configuration are presented in Figure 2. Figure 2a illustrates the polyurea curing reaction between the isocyanate-containing and amine-containing components, and Figure 2b shows the representative urea linkage formed in the cured polyurea network. This curing structure provides the basis for forming the polymer matrix that holds the R.T.P. and silica sand. Figure 2c shows the microscopic image of the R.T.P. used in this study. The R.T.P. consisted of numerous spherical or quasi-spherical microcapsule particles with micrometer-scale morphology, suggesting that the pigment has a particle morphology suitable for dispersion within the polyurea matrix. Figure 2d schematically illustrates the core–shell structure of the R.T.P., in which the outer shell protects the internal thermochromic components and the core provides the temperature-responsive color-changing function. Figure 2e presents the conceptual configuration of the final composite consisting of aliphatic polyurea resin, R.T.P., and silica sand. In the composite, the polyurea resin acts as the continuous matrix, the R.T.P. acts as the functional particles responsible for low-temperature color development, and the silica sand acts as the inorganic filler.

### 2.2. Mix Design and Preparation of Polymer Composites

In this study, temperature-responsive polymer composites were prepared by mixing aliphatic polyurea resin, R.T.P., and silica sand. The main mixture variables were the binder content and R.T.P. dosage. The binder contents were set to 8, 10, 12, and 14 wt.%, and the R.T.P. dosages were set to 10 and 20 phr. The binder and silica sand contents were expressed as wt.% of the binder–sand base mixture, whereas the R.T.P. dosage was expressed in phr relative to the total binder mass. The silica sand contents were adjusted to 92, 90, 88, and 86 wt.% according to the binder content. The resin component and hardener of the aliphatic polyurea resin were mixed at a mass ratio of 5:5. The detailed mixture proportions are summarized in Table 5.

The composites were prepared using the same mixing procedure for all mixture conditions. First, the silica sand was weighed according to the target mixture proportion and placed in a mixing bowl. The resin component and hardener of the aliphatic polyurea resin were then weighed at a mass ratio of 5:5 and premixed at 800 rpm for 1 min. Because direct dry mixing of R.T.P. with silica sand could cause airborne dispersion and loss of the fine pigment particles, the R.T.P. was first dispersed in the polyurea binder. The R.T.P.-dispersed binder was then added to the mixing bowl containing the silica sand, and the entire mixture was mixed until a visually uniform composite mixture was obtained. This mixing procedure was adopted to reduce pigment loss during preparation and to improve the dispersion of R.T.P. within the binder phase. The same procedure was applied to all mixtures to enable comparison of the effects of binder content and R.T.P. dosage on low-temperature color response and basic mechanical performance.

### 2.3. Specimen Preparation and Conditioning

The prepared temperature-responsive polyurea-based composites were cast into different specimen geometries according to the evaluation purpose. For the evaluation of color change and colorimetric characteristics under low-temperature conditions, disk-shaped specimens with a diameter of 90 mm and a thickness of 10 mm were fabricated. The disk-shaped geometry was selected to provide a uniform surface shape and thickness, enabling observation of surface color development, color uniformity, and colorimetric changes caused by temperature variation.

For mechanical performance evaluation, prismatic specimens with dimensions of 40 mm × 40 mm × 160 mm were fabricated. These specimens were used for compressive strength and flexural strength tests to examine the basic strength, fracture resistance, and structural stability required for preliminary pavement filling applications. The specimen geometry was adopted from the prismatic specimen dimensions specified in ASTM C348-14 for flexural strength testing of hydraulic-cement mortars [53]. However, because the material investigated in this study was a polyurea-based composite rather than a hydraulic-cement mortar, the mortar-specific preparation and flow requirements were not applied.

**Figure 3:**
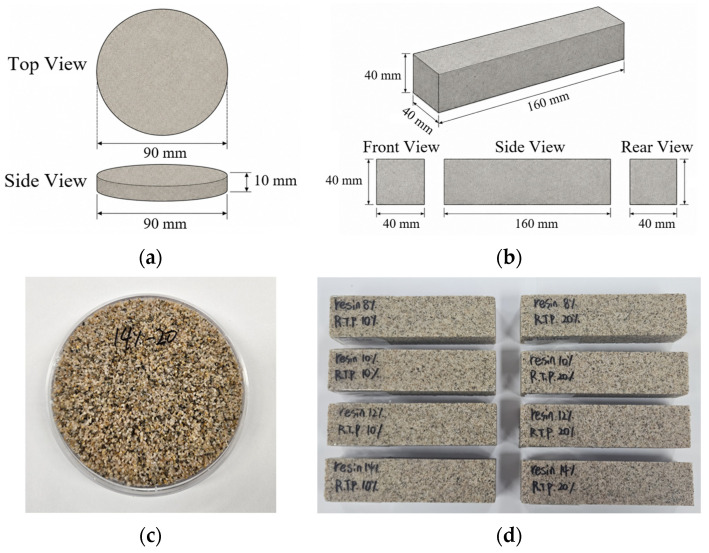
Geometry and appearance of thermochromic polyurea-based composite specimens: (**a**) circular specimen for colorimetric characterization, (**b**) prismatic specimen for mechanical characterization, (**c**) fabricated circular specimen, and (**d**) fabricated prismatic specimen.

All specimens were demolded 24 h after casting and then conditioned for 7 days under controlled conditions of 23 ± 2 °C and 65 ± 5% relative humidity. After conditioning, the disk-shaped specimens were used for colorimetric evaluation, and the prismatic specimens were used for compressive and flexural strength tests. The specimen geometries and fabricated specimens are shown in Figure 3.

### 2.4. Colorimetric Characterization

Colorimetric analysis was performed to quantitatively evaluate the low-temperature color response of the temperature-responsive polyurea-based composites. Color measurements were conducted using a spectrophotometer CM-25cG (Konica Minolta, Tokyo, Japan) [54]. The measurement conditions were set to standard illuminant D65 and a 10° standard observer. The color measurement was performed at the center of each disk-shaped specimen, and the measured value was used as the representative colorimetric value of the specimen.

Color measurements were conducted under room-temperature and low-temperature conditions. A non-thermochromic control specimen without R.T.P. was measured at 20 °C as the reference color, whereas the R.T.P.-containing specimens were measured after inducing color development under low-temperature conditions. The low-temperature condition was produced using a temperature–humidity chamber, and the chamber temperature was set to −10 °C. The specimens were placed in the chamber at 20 °C and allowed to stabilize for approximately 5 h, during which the specimen temperature decreased to approximately −6 °C before color measurement. During low-temperature measurement, the surface temperature of the specimens was separately checked using an infrared thermometer (Daekwang, Inc., Seoul, Republic of Korea).

The measured color values were organized using the CIE $L^{*}a^{*}b^{*}$ color space. In the CIE $L^{*}a^{*}b^{*}$ color space, $L^{*}$ represents lightness, $a^{*}$ represents the red–green axis, and $b^{*}$ represents the yellow–blue axis. In addition, to visually compare the color distribution according to R.T.P. dosage and binder content, the measured CIE $L^{*}a^{*}b^{*}$ values were converted into CIE XYZ tristimulus values, and the CIE 1931 x, y chromaticity coordinates were then calculated.

To calculate the CIE XYZ values from the CIE $L^{*}a^{*}b^{*}$ values, the reference white values for the D65/10° condition were applied. The reference white values used in this study were $X_{n}=94.811$, $Y_{n}=100.000$, and $Z_{n}=107.304$. First, $f_{x}$, $f_{y}$, and $f_{z}$ were calculated from the $L^{*}$, $a^{*}$, and $b^{*}$ values as Equations (1)–(3):

(1) $$\begin{array}{c} f_{y}=\frac{{(L}^{*}+16)}{116} \end{array}$$

(2) $$\begin{array}{c} f_{x}=f_{y}+(\frac{a^{*}}{500}) \end{array}$$

(3) $$\begin{array}{c} f_{z}=f_{y}-(\frac{b^{*}}{200}) \end{array}$$

The CIE XYZ tristimulus values were then calculated using the following Equations (4)–(6):

(4) $$\begin{array}{c} X=X_{n}\times {f_{x}}^{3} \end{array}$$

(5) $$\begin{array}{c} Y=Y_{n}\times {f_{y}}^{3} \end{array}$$

(6) $$\begin{array}{c} Z=Z_{n}\times {f_{z}}^{3} \end{array}$$

Finally, the CIE 1931 x, y chromaticity coordinates were calculated from the X, Y, and Z values as Equations (7) and (8):

(7) $$\begin{array}{c} x=\frac{X}{X+Y+Z} \end{array}$$

(8) $$\begin{array}{c} y=\frac{Y}{X+Y+Z} \end{array}$$

The color difference between the control specimen and each R.T.P.-containing specimen was calculated using the CIE 1976 color-difference equation. The control specimen was used as the reference because it represents the non-thermochromic composite without R.T.P. The color difference, ${∆E}_{ab}^{*}$, was used as a quantitative indicator of the visual contrast produced by the thermochromic composite under the low-temperature condition (Equation (9)).

(9) $$\begin{array}{c} {∆E}_{ab}^{*}=\sqrt{{\left(L_{i}^{*}-L_{c}^{*}\right)}^{2}+{\left(a_{i}^{*}-a_{c}^{*}\right)}^{2}+{\left(b_{i}^{*}-b_{c}^{*}\right)}^{2}} \end{array}$$

where $L_{i}^{*}$, $a_{i}^{*}$, and $b_{i}^{*}$ are the color coordinates of an R.T.P.-containing specimen, and $L_{c}^{*}$, $a_{c}^{*}$, and $b_{c}^{*}$ are the color coordinates of the control specimen.

**Figure 4:**
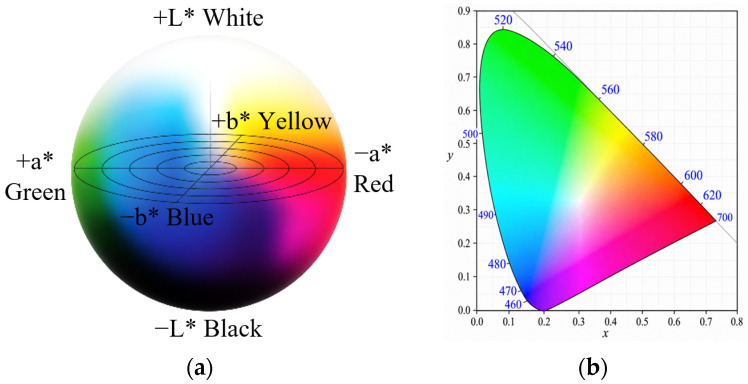
Colorimetric characterization of temperature-responsive polyurea-based composites: (**a**) CIE $L^{*}a^{*}b^{*}$ color space, and (**b**) CIE 1931 x, y chromaticity diagram.

Using this procedure, the low-temperature color response of each mixture was compared in both the CIE $L^{*}a^{*}b^{*}$ color space and the CIE 1931 x, y chromaticity diagram. The CIE $L^{*}a^{*}b^{*}$ color space was used to evaluate changes in lightness and color axes among the specimens, whereas the CIE 1931 x, y chromaticity diagram was used to examine the distribution of chromaticity coordinates and the direction of color shift. The colorimetric analysis concepts are shown in Figure 4.

### 2.5. Mechanical Characterization

Compressive and flexural strength tests were conducted using a universal testing machine (UH-1000KNI, Shimadzu Corporation, Kyoto, Japan) [55]. The compressive strength test was conducted with reference to ASTM C349-18, Standard Test Method for Compressive Strength of Hydraulic-Cement Mortars [56], and the flexural strength test was conducted with reference to ASTM C348-14, Standard Test Method for Flexural Strength of Hydraulic-Cement Mortars [53]. Although these standards are intended for hydraulic-cement mortars, they were adopted as reference methods in this study to maintain consistent specimen geometry, loading configuration, and strength calculation for preliminary comparative screening of the groove-filling polyurea-based mixtures.

For each mixture condition, the flexural strength test was performed using three prismatic specimens. Each specimen was placed on a three-point bending fixture, and load was applied until failure. The maximum load measured at failure was used to calculate the maximum flexural moment. After the flexural strength test, the two broken halves of each prism were used for the compressive strength test. Therefore, the flexural strength of each mixture was calculated from three measurements, whereas the compressive strength was calculated from six measurements. The mean value and standard deviation were then calculated for each mixture condition. The compressive strength was calculated using the maximum load measured at failure, as expressed in Equation (10):

(10) $$\begin{array}{c} f_{c}=\frac{P}{A} \end{array}$$

where $f_{c}$ is the compressive strength (MPa), P is the maximum load applied to the specimen (N), and A is the loaded area (mm^2^).

**Figure 5:**
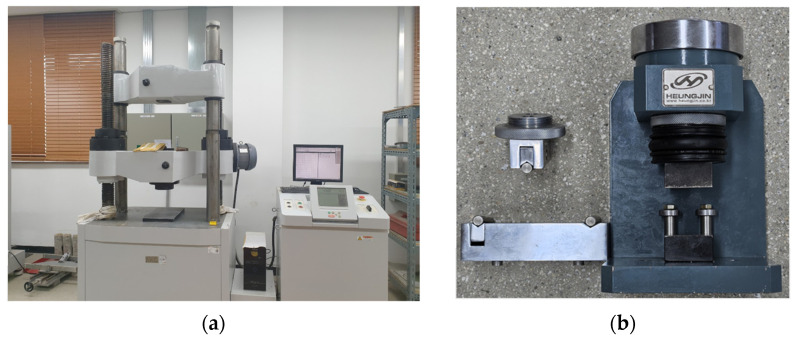
Mechanical characterization test setup: (**a**) compressive strength test fixture and (**b**) flexural strength test fixture.

For flexural strength calculation, the maximum flexural moment in the three-point bending test was calculated as Equation (11):

(11) $$\begin{array}{c} M=\frac{PL}{4} \end{array}$$

where M is the maximum flexural moment (N·mm), P is the maximum load measured at failure (N), and L is the span length (mm). In this study, the span length for the flexural strength test was set to 100 mm. The flexural strength was then calculated as Equation (12):

(12) $$\begin{array}{c} f_{b}=\frac{6M}{bd^{2}} \end{array}$$

where $f_{b}$ is the flexural strength (MPa), b is the specimen width (mm), and d is the specimen depth (mm). In this study, the compressive strength was used to evaluate the basic load-bearing resistance of the composites according to mixture condition, while the flexural strength was used to examine flexural load resistance and fracture resistance of the composites. The mechanical test setup and fixtures used for the evaluation are shown in Figure 5.

### 2.6. Preliminary Groove-Filling Applicability Test

A preliminary groove-filling applicability evaluation was conducted to examine the potential use of the temperature-responsive polyurea-based composite on pavement surfaces. This evaluation was performed as a preliminary filling applicability assessment to determine whether the composite could be physically placed into grooves formed on a concrete surface.

A groove-simulated concrete panel was prepared using a 300 mm × 300 mm concrete block. The groove geometry was determined with reference to the runway pavement grooving configuration specified in FAA AC 150/5320-12C [57]. In this study, a laboratory-scale groove-simulated pavement surface was prepared with a groove width of 6.0 mm, groove depth of 6.0 mm, and center-to-center spacing of 38.0 mm. The grooves were formed using a concrete cutter, and the groove geometry and surface condition were visually checked before filling.

Before placing the composite mixture, the grooves and concrete surface were cleaned using a blower to remove residual dust and foreign materials. This step was performed because residual dust and debris can affect the filling behavior and surface adhesion of polymer-based mixtures. No primer was used in this preliminary evaluation, because the primary purpose was to examine the physical filling capability of the composite into the groove space. The polyurea-based composite was then placed into the grooves, and repeated rolling and surface-filling operations were performed to help the mixture flow into the groove space. After filling, the groove-filled panel was left to cure for approximately 1 h before the low-temperature color response was examined.

**Figure 6:**
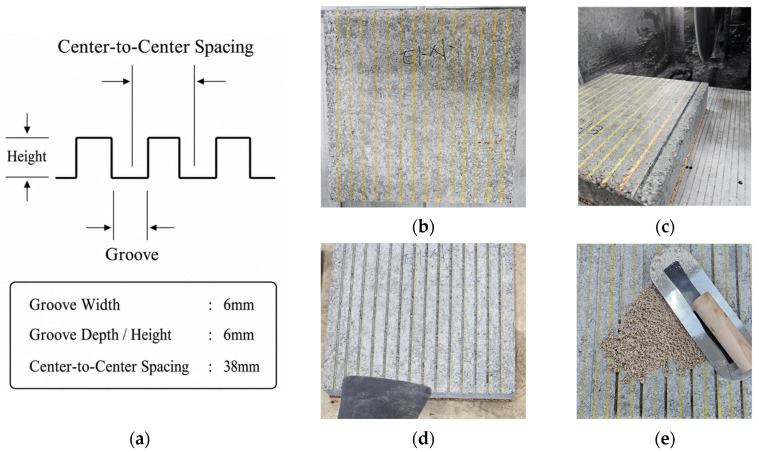
Preliminary groove-filling applicability test based on the FAA grooving configuration: (**a**) groove geometry and dimensions of the groove-simulated concrete panel, (**b**) concrete block before grooving, (**c**) groove formation using a concrete cutter, (**d**) surface cleaning with a blower, and (**e**) groove filling and rolling of the polymer composite.

This evaluation was conducted as a preliminary test to confirm the groove-filling feasibility of the polyurea-based composite under laboratory-scale conditions. The groove-simulated concrete panel and filling procedure are shown in Figure 6.

## 3. Results

### 3.1. FE-SEM Observation of the Cross-Sectional Morphology of the Thermochromic Polyurea-Based Composite

**Figure 7:**
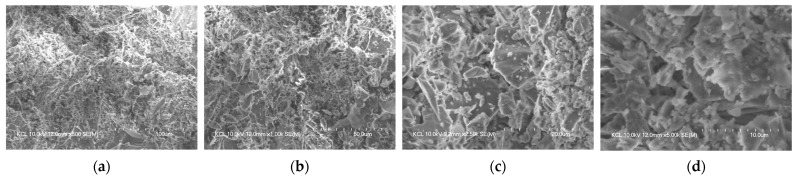
FE-SEM images of the cross-sectional morphology of the thermochromic polyurea-based composite at different magnifications: (**a**) 500×, (**b**) 1000×, (**c**) 2500×, and (**d**) 5000×.

To provide microstructural evidence for the proposed composite, the cross-sectional morphology of the final thermochromic polyurea-based composite was observed using field-emission scanning electron microscopy (FE-SEM; S-4700, Hitachi, Tokyo, Japan). The observed composite consisted of aliphatic polyurea resin, R.T.P., and silica sand. FE-SEM images were obtained at magnifications of 500×, 1000×, 2500×, and 5000×, as shown in Figure 7.

At lower magnifications of 500× and 1000×, the cross-section showed a heterogeneous but continuous composite morphology, indicating that particulate phases were embedded within the cured polyurea matrix. At higher magnifications of 2500× and 5000×, rough surface features and particle-embedded regions were more clearly observed. These features are consistent with the intended multi-phase structure of the thermochromic polyurea-based composite, supporting that the proposed material was formed as a bulk composite rather than as a simple surface-applied layer.

### 3.2. Low-Temperature Color Response of Thermochromic Polyurea-Based Composites

**Figure 8:**
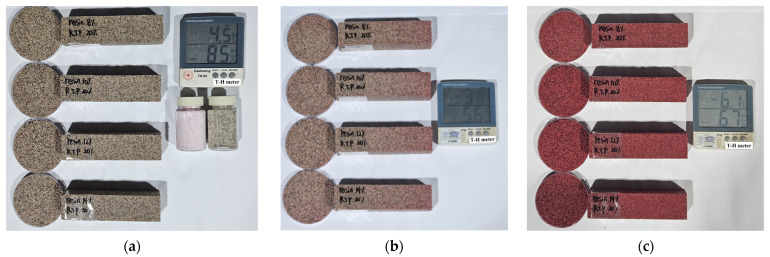
Visual color response of thermochromic polyurea-based composite specimens under different temperature conditions: (**a**) 4.5 °C, (**b**) −3.8 °C, and (**c**) −6.1 °C.

The surface color change of the thermochromic polyurea-based composite specimens was compared under room-temperature and low-temperature conditions to evaluate the low-temperature color response. The non-thermochromic control specimen was measured at 20 °C, whereas the R.T.P.-containing specimens were observed during cooling. Figure 8 shows the visual color response of the thermochromic specimens under different temperature conditions.

Visual observation showed that distinct red color development was not observed at 4.5 °C. However, a reddish color began to appear at approximately −3.8 °C, and the most distinct color development was observed when the specimen temperature decreased to approximately −6.1 °C. Compared with the coloration temperature range of the R.T.P. powder, the onset of visible color change in the composite specimens was observed at a temperature approximately 1.3 °C lower than the lower limit of the reported coloration range, −2.5 °C. The most distinct color development was observed at −6.1 °C, which was approximately 3.6 °C lower than this lower limit. This result indicates that, when R.T.P. is incorporated into a polyurea binder and silica sand composite, the actual color-development behavior of the composite can differ from the nominal transition range of the pigment alone. In particular, the red color development was generally more distinct in the mixtures containing 20 phr R.T.P. than in those containing 10 phr R.T.P., suggesting that increasing the thermochromic pigment dosage can enhance the low-temperature visual indication of the composite.

The quantitative colorimetric results were consistent with the visual observations. Table 6 presents the CIE $L^{*}a^{*}b^{*}$ values, CIE 1931 x, y chromaticity coordinates, and color difference ${∆E}_{ab}^{*}$ values of the control and thermochromic polyurea-based composite specimens. The $a^{*}$ value of the control specimen was 3.85 at 20 °C, whereas the R.T.P.-containing specimens showed higher positive $a^{*}$ values under the representative low-temperature condition of approximately −6.1 °C. In addition, all R.T.P.-containing specimens showed ${∆E}_{ab}^{*}$ values of 17.06–28.17 relative to the control specimen, confirming that the incorporation of R.T.P. produced a clear color difference under the low-temperature condition. In particular, Specimen 6, with a binder content of 10 wt.% and an R.T.P. dosage of 20 phr, showed the highest $a^{*}$ value of 22.95 and a relatively high ${∆E}_{ab}^{*}$ value of 22.12, indicating pronounced red color development with clear visual contrast.

An increase in R.T.P. dosage was generally accompanied by a decrease in $L^{*}$ value. This indicates that red color development was enhanced under low-temperature conditions while the surface lightness decreased. Therefore, the increase in $a^{*}$ value together with the decrease in $L^{*}$ value suggests that the color change can become more visually distinguishable to drivers or road managers under low-temperature conditions.

**Figure 9:**
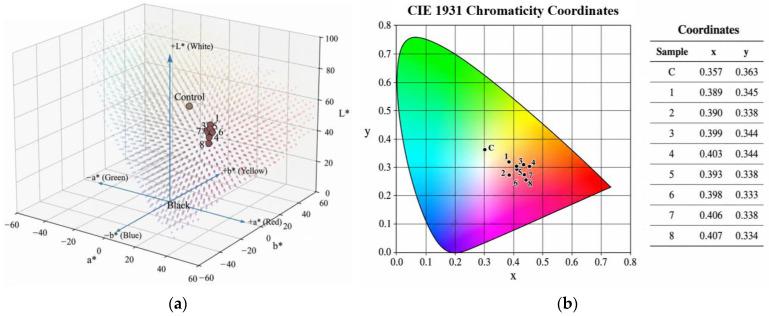
Sample positions of control and thermochromic polyurea-based composite specimens in color spaces: (**a**) CIE $L^{*}a^{*}b^{*}$ color space and (**b**) CIE 1931 x, y chromaticity diagram.

Figure 9 shows the colorimetric results from Table 6 plotted in the CIE $L^{*}a^{*}b^{*}$ color space and CIE 1931 x, y chromaticity diagram. In the CIE $L^{*}a^{*}b^{*}$ color space, the R.T.P.-containing specimens shifted toward the positive $a^{*}$ direction compared with the control specimen, confirming enhanced red color development under low-temperature conditions. In the CIE 1931 x, y chromaticity diagram, the chromaticity coordinates of the thermochromic specimens were distributed in a region distinguishable from that of the control specimen, indicating that the low-temperature color change was also identifiable in the chromaticity coordinate system.

The effect of binder content on color development did not show a consistently increasing trend. This may be attributed to simultaneous changes in pigment dispersion, pigment exposure at the specimen surface, and the optical transparency of the resin matrix as the binder content changed. Under the 20 phr R.T.P. condition, the mixture with 10 wt.% binder content showed the highest $a^{*}$ value, suggesting that this mixture provided a relatively balanced condition for pigment dispersion and surface color development. Based on the low-temperature color response, Specimen 6, with 10 wt.% binder content and 20 phr R.T.P., showed the most pronounced colorimetric response among the tested mixtures.

### 3.3. Mechanical Properties of Thermochromic Polyurea-Based Composites

**Figure 10:**
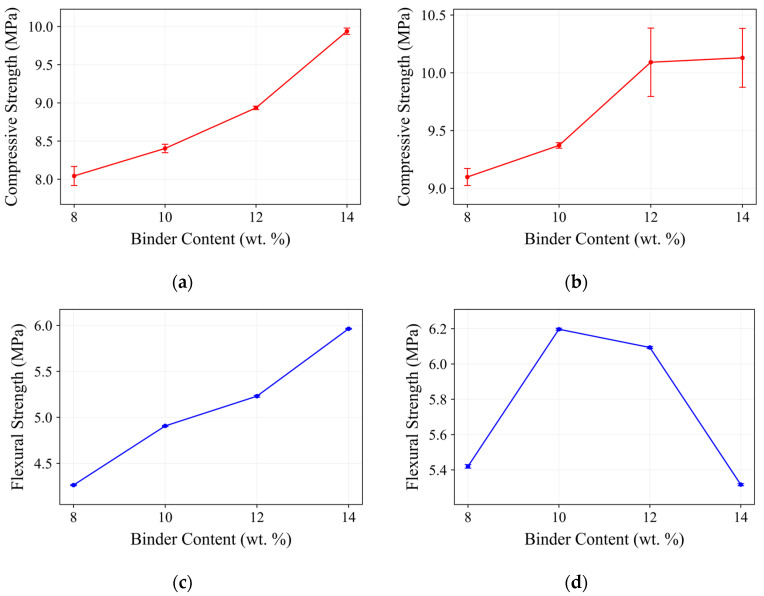
Mechanical strength variation of thermochromic polyurea-based composites according to binder content and R.T.P. dosage: (**a**) compressive strength at R.T.P. 10 phr, (**b**) compressive strength at R.T.P. 20 phr, (**c**) flexural strength at R.T.P. 10 phr, and (**d**) flexural strength at R.T.P. 20 phr.

The compressive and flexural strengths of the thermochromic polyurea-based composites were evaluated to examine their basic mechanical performance for preliminary pavement filling applications. Table 7 presents the mean compressive and flexural strengths and standard deviations for each mixture condition. The flexural strength was calculated from three prismatic specimens for each mixture, and the compressive strength was calculated from six broken halves obtained after flexural testing. Figure 10 compares the average compressive and flexural strengths according to binder content and R.T.P. dosage.

The compressive strength generally increased with increasing binder content. Under the 10 phr R.T.P. condition, the average compressive strength increased from 8.04 MPa to 9.94 MPa as the binder content increased from 8 wt.% to 14 wt.%. Under the 20 phr R.T.P. condition, the average compressive strength also increased from 9.10 MPa to 10.13 MPa. This tendency indicates that increasing the binder content improved the basic load-bearing resistance of the composite within the investigated mixture range.

At the same binder content, the mixtures containing 20 phr R.T.P. generally showed higher compressive strength than those containing 10 phr R.T.P. For example, at a binder content of 8 wt.%, the average compressive strengths of the 10 phr and 20 phr R.T.P. mixtures were 8.04 MPa and 9.10 MPa, respectively. At a binder content of 12 wt.%, the corresponding values were 8.94 MPa and 10.09 MPa. These results indicate that increasing the R.T.P. dosage from 10 phr to 20 phr did not reduce the compressive strength of the composite in the tested range.

The flexural strength showed a different tendency from the compressive strength. Under the 10 phr R.T.P. condition, the average flexural strength continuously increased from 4.26 MPa to 5.96 MPa as the binder content increased from 8 wt.% to 14 wt.%. In contrast, under the 20 phr R.T.P. condition, the highest average flexural strength of 6.20 MPa was obtained for Specimen 6 with 10 wt.% binder content. When the binder content increased to 12 wt.% and 14 wt.%, the average flexural strength decreased to 6.09 MPa and 5.32 MPa, respectively.

Among all mixtures, Specimen 8, containing 14 wt.% binder and 20 phr R.T.P., showed the highest average compressive strength of 10.13 MPa. However, Specimen 6, containing 10 wt.% binder and 20 phr R.T.P., showed the highest average flexural strength of 6.20 MPa. Considering that preliminary pavement filling applications require both basic load-bearing resistance and resistance to flexural cracking, the flexural strength result is important for evaluating the mechanical suitability of the composite.

Together with the colorimetric results in Section 3.2, Specimen 6 showed the most balanced performance among the tested mixtures. This mixture exhibited the highest $a^{*}$ value in the low-temperature colorimetric evaluation and the highest average flexural strength in the mechanical evaluation, while maintaining a compressive strength of 9.37 MPa. Therefore, Specimen 6 was selected as the candidate mixture for the subsequent preliminary groove-filling applicability test.

### 3.4. Preliminary Groove-Filling Applicability

**Figure 11:**
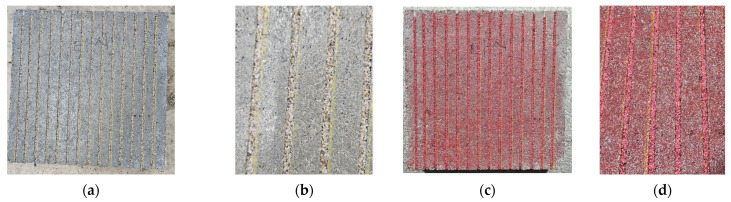
Groove-filling applicability and color response of the selected thermochromic composite: (**a**) overall view at room temperature, (**b**) close-up view at room temperature, (**c**) overall view under low-temperature condition, and (**d**) close-up view under low-temperature condition.

Based on the colorimetric and mechanical evaluation results, Specimen 6, with 10 wt.% binder content and 20 phr R.T.P., was used for the preliminary groove-filling applicability test. Figure 11 shows the application of the selected temperature-responsive polyurea-based composite to the groove-simulated concrete panel. When the mixture was placed into the grooves without primer, relatively low initial adhesion to the concrete surface was observed during placement. However, after repeated rolling and surface-filling operations, the mixture was introduced into the grooves and was arranged relatively continuously along the groove lines. After filling, no obvious unfilled regions were observed inside the grooves, and the filled grooves showed generally acceptable surface appearance without excessive protrusion.

The low-temperature color response of the filled composite was also confirmed after groove filling. Under low-temperature conditions, a distinct reddish color change was observed in the composite placed inside the grooves. In both the overall top-view image and the magnified image, continuous red color development appeared along the filled groove lines. This result indicates that the low-temperature color response observed in the disk-shaped specimens can be maintained when the composite is applied inside groove structures.

These results show that the selected composite can be physically placed into pavement grooves and can retain its thermochromic color response in a groove-filled state. Therefore, the groove-filling approach has potential as a localized application method for positioning the thermochromic composite on pavement surfaces. However, this evaluation was conducted as a laboratory-scale preliminary test using a small groove-simulated concrete panel, rather than under actual road construction conditions. During repeated rolling, part of the mixture spread onto the concrete surface outside the grooves, resulting in color development not only inside the grooves but also on the surrounding surface. For practical pavement application, further process optimization is required to selectively fill the grooves and minimize surface residue.

### 3.5. Overall Evaluation and Candidate Mix Selection

To select a candidate mixture for the temperature-responsive polyurea-based composite, the low-temperature color response, compressive strength, and flexural strength were comprehensively evaluated. Because the purpose of the composite is to visually indicate low-temperature pavement conditions associated with black ice risk, the candidate mixture was selected by considering not only mechanical strength but also color development under low-temperature conditions. Table 8 summarizes the main comparative evaluation items and the most favorable specimen for each criterion.

In terms of low-temperature color response, the mixtures containing 20 phr R.T.P. generally showed more distinct red color development than those containing 10 phr R.T.P. Among all mixtures, Specimen 6 showed the highest $a^{*}$ value of 22.95, indicating the most pronounced red color development under low-temperature conditions. This result suggests that Specimen 6 is advantageous in terms of visual color response for indicating black ice-prone pavement conditions.

In terms of mechanical performance, the compressive strength generally increased with increasing binder content, and Specimen 8 showed the highest average compressive strength of 10.13 MPa. However, the average flexural strength of Specimen 8 was 5.32 MPa, which was lower than that of Specimen 6. Specimen 6 showed the highest average flexural strength of 6.20 MPa while maintaining a compressive strength of 9.37 MPa. Although Specimen 8 showed the highest ${∆E}_{ab}^{*}$ value and compressive strength, its higher color difference was mainly associated with reduced lightness rather than the strongest red color development.

Considering both the colorimetric response and mechanical performance, Specimen 6, with 10 wt.% binder content and 20 phr R.T.P., was selected as the most balanced candidate mixture in this study. This mixture showed the highest $a^{*}$ value, the highest average flexural strength, and a comparable compressive strength within the tested range. The subsequent preliminary groove-filling applicability test confirmed that the selected mixture could be physically placed into the groove-simulated concrete panel and could retain its low-temperature color response in the filled state.

## 4. Discussion

This study evaluated the preliminary feasibility of thermochromic polyurea-based composites containing reversible thermochromic pigment by examining their low-temperature color response, basic mechanical performance, and laboratory-scale groove-filling applicability. The proposed composite was designed to provide a material-based visual indication of low-temperature pavement conditions associated with black ice formation by converting a decrease in pavement temperature into a visible color response.

From a materials-design perspective, the effectiveness of the proposed composite depends on the balance among the polyurea matrix, R.T.P. microcapsules, and silica sand filler. Dispersing R.T.P. in the aliphatic polyurea binder before adding silica sand is important because the binder phase governs pigment fixation, optical transparency, and temperature-responsive color expression while reducing pigment loss during mixing. At the same time, silica sand contributes to volumetric stability and basic mechanical resistance, but its content can also influence pigment exposure and surface lightness. Therefore, the color response and mechanical performance should be interpreted as coupled material responses controlled by pigment dosage, binder continuity, and filler packing rather than by R.T.P. dosage alone. The FE-SEM observation provides qualitative microstructural support for this pigment–binder–filler mixture design. The cross-sectional images showed a heterogeneous but continuous bulk morphology, with particulate phases embedded within the cured polyurea matrix. This morphology is consistent with the intended composite configuration, in which the polyurea phase serves as the matrix for incorporating thermochromic pigment and silica sand filler. However, because FE-SEM mainly provides morphological information, the observation was interpreted as qualitative microstructural evidence rather than quantitative chemical identification.

The color-development behavior of the polyurea-based composite did not exactly follow the nominal coloration temperature range of the R.T.P. powder. This difference is reasonable because the pigment was not used as an isolated powder but was incorporated into a composite system consisting of polyurea binder and silica sand. In this form, the actual color response can be affected by heat-transfer conditions, pigment dispersion, pigment exposure at the specimen surface, optical transparency of the binder, and interactions with the inorganic filler. Therefore, when thermochromic pigments are applied to pavement materials, the transition temperature of the pigment alone is not sufficient to evaluate the practical color response. The color-development behavior should be examined under the actual mixture and application conditions.

The colorimetric results confirmed that the R.T.P.-containing composites developed a reddish color under low-temperature conditions. This response was quantitatively reflected by an increase in the CIE $a^{*}$ value. The R.T.P.-containing mixtures showed ${∆E}_{ab}^{*}$ values of 17.06–28.17 relative to the non-thermochromic control specimen, indicating measurable color contrast within the present composite system. For the selected mixture, the $L^{*}$ value decreased by 10.54 and the $a^{*}$ value increased by 19.10 compared with the control specimen, confirming pronounced red color development with reduced surface lightness. For visual warning applications, this contrast is important because black ice-prone pavement conditions are difficult to distinguish from wet pavement surfaces. A clear color change on the pavement surface may therefore support visual recognition of low-temperature hazardous conditions by drivers or road managers.

However, the color response cannot be optimized only by increasing the R.T.P. dosage. Although a higher pigment dosage can enhance low-temperature color development, it may also affect mixture uniformity, pigment dispersion, workability, and mechanical performance. The mechanical test results supported the need for balanced mixture design. The compressive strength generally increased with increasing binder content, indicating improved bonding among silica sand particles. In contrast, the flexural strength did not increase in the same manner. Under the 20 phr R.T.P. condition, the highest flexural strength was obtained at 10 wt.% binder content. This suggests that excessive binder content is not necessarily advantageous and that pigment dispersion, particle interaction, stress transfer, and continuity of the resin matrix can collectively affect flexural performance. Therefore, the mixture with 10 wt.% binder content and 20 phr R.T.P. was selected not simply because of the strongest color response, but because it provided the most balanced combination of colorimetric response and mechanical performance.

The preliminary groove-filling test showed that the selected composite could be physically placed into groove structures and could retain its low-temperature color response in the groove-filled state. This result suggests that the proposed composite may be applied as a localized functional material positioned within pavement grooves rather than as a full-area surface coating. Locating the material inside grooves may partially reduce direct tire contact and wear exposure, although this potential advantage should be verified through further durability testing. Such localized groove-filling application may be useful for black ice-prone areas such as bridge decks, elevated roads, tunnel entrances and exits, shaded sections, mountain roads, and ramp sections, where selective placement within targeted groove areas could reduce the amount of functional material required compared with application over larger pavement surface areas.

Several limitations should be considered. First, this study evaluated color development under low-temperature conditions but did not directly verify actual black ice formation. Because black ice formation is governed by pavement temperature, surface moisture, ambient humidity, and solar radiation, the influence of these environmental conditions should be additionally considered when interpreting the color response of the proposed composite. Second, thermochromic pigments and polymer binders can be affected by ultraviolet radiation, oxygen, moisture, deicing salts, repeated freeze–thaw cycles, and tire-induced wear. Therefore, repeated thermochromic cycling, UV stability, abrasion resistance, adhesion strength, and freeze–thaw durability should be evaluated. Third, the groove-filling test was conducted using a small laboratory-scale concrete panel. For practical pavement application, the effects of groove width, depth, spacing, filling amount, surface preparation, drying condition, primer application, and finishing process should be further investigated. During the preliminary filling test, part of the mixture spread onto the concrete surface outside the grooves, indicating that viscosity control, filling amount control, surface masking, scraping, or a dedicated finishing device may be required to minimize surface residue. In addition, the visual effectiveness of the thermochromic color response should be assessed under realistic road-viewing conditions. Future studies should consider recognition distance, illumination conditions, viewing angle, response time, visual perception, and driver behavior to determine whether the color change can be effectively recognized as a warning cue in real driving situations.

Overall, the results indicate that thermochromic polyurea-based composites containing R.T.P. can provide visible color change under low-temperature conditions while maintaining basic mechanical performance and preliminary groove-filling applicability. In particular, the mixture with 10 wt.% binder content and 20 phr R.T.P. was selected as the most balanced candidate mixture within the tested range by considering the binder characteristics, reversible thermochromic response of R.T.P., temperature-dependent visibility, and both compressive and flexural strengths. These findings demonstrate the potential of the proposed thermochromic polyurea-based composite as a responsive pavement material that can provide visible surface information for improving winter road safety.

## 5. Conclusions

This study evaluated the preliminary feasibility of a groove-filling thermochromic polyurea-based composite as a material-based visual indicator for low-temperature pavement conditions associated with black ice risk. For this purpose, the polymer binder was selected, and the effects of R.T.P. dosage and binder content on colorimetric response, compressive strength, flexural strength, and preliminary groove-filling applicability were investigated. The main conclusions are as follows:

- The visible color-change onset of the composite occurred approximately 1.3 °C lower than the lower limit of the nominal coloration range of the R.T.P. powder. This result suggests that the actual color-development behavior can shift when thermochromic pigment is incorporated into a composite system.
- The R.T.P.-containing composites showed ${∆E}_{ab}^{*}$ values of 17.06–28.17 relative to the non-thermochromic control specimen. For the selected mixture, the $L^{*}$, $a^{*}$, and $b^{*}$ values were 47.82, 22.95, and 8.52, respectively, indicating pronounced red color development under the low-temperature condition.
- The composites maintained compressive and flexural strengths of up to 10.13 ± 0.26 MPa and 6.20 ± 0.01 MPa, respectively. The selected mixture showed 9.37 ± 0.02 MPa in compressive strength and 6.20 ± 0.01 MPa in flexural strength.
- The preliminary groove-filling test confirmed that the thermochromic polyurea-based composite could be used as a localized groove-filling material for black ice-prone pavement sections by retaining its low-temperature color response in the filled state.

Overall, this study demonstrated the potential of thermochromic polyurea-based composites as material-based passive visual indicators for low-temperature pavement conditions associated with black ice risk. Considering both low-temperature color response and mechanical performance, the mixture containing 10 wt.% aliphatic polyurea binder and 90 wt.% silica sand in the binder–sand base mixture, with R.T.P. added at 20 phr relative to the binder mass, was selected as the most balanced candidate mixture within the tested range. Field validation of repeated thermochromic cycling stability, ultraviolet exposure stability, adhesion strength, abrasion resistance, freeze–thaw durability, deicing salt resistance, and performance under actual traffic loading conditions will further clarify the practical applicability of the proposed composite. Through such validation, the thermochromic polyurea-based composite may contribute to winter road safety by providing visible pavement-surface information in black ice-prone areas.

## Figures and Tables

**Table 1:** Summary of literature review.

Author (Year)	Purpose	Methods	Results	Limitation
Zhan et al. (2023) [43]	Low-temperature visual indicator	Au-LCE bilayer membrane; crack-opening mechanism; CIE 1931 chromaticity analysis	Visual signal generated under temperature decrease; onset temperature tunable by material design	Not a pavement material; no road-surface durability or traffic-loading evaluation
Hu et al. (2015) [45]	Thermochromic asphalt binder for pavement temperature regulation	Thermochromic powders in PG64-22 asphalt binder; spectral reflectance and Superpave binder performance tests	Changes in penetration, complex modulus, rutting/fatigue parameters, and high-temperature binder performance	Binder-level study; not focused on low-temperature visual warning
Hu and Yu (2016) [46]	Thermochromic asphalt coating for seasonal thermal control	Thermochromic asphalt coating; thermal, optical, chemical, and outdoor temperature analyses	Surface-temperature reductions of 6.6 °C, 2.7 °C, and 4.9 °C for black, blue, and red coatings, respectively	Coating-based thermal management; limited connection to black ice-prone visual warning
Chen et al. (2021) [47]	UV-aging durability of thermochromic bitumen	Accelerated UV aging; rheological, chemical, and microstructural analyses	30 days of indoor UV aging was approximately equivalent to 50 months of outdoor solar radiation aging	Not designed for black ice-prone visual indication or pavement-surface composite application
Zhang et al. (2022) [48]	Thermochromic pavement coating for dynamic thermoregulation	Red/black TCM coatings; spectral reflectance, CIE L*a*b*, thermal test, and CFD simulation	Optimal TiO_2_ contents were 2 wt.% for red TCM and 5 wt.% for black TCM; red TCM coating achieved a cooling effect of 10.57 °C	Focused on UHI mitigation; road-service durability and optical aging remained future work
Li et al. (2023) [49]	Composite coating for cooling and ice suppression	Reflectance, light aging, ice-melting, skid resistance, and road performance tests	Optimal TCM-black TiO_2_ content was 3 wt.%; TCM-top/SMIS-bottom double layer achieved 9 °C cooling	Not designed as a passive thermochromic warning composite
Chen et al. (2025) [50]	Red thermochromic coating for pavement cooling	Thermochromic red/silicon iron red composite coating; UV–Vis–NIR, CIE L*a*b*, SEM, TGA	Optimized ratio: 75% Thermochromic Red + 25% Silicon Iron Red; NIR reflectance 71.36%; visible reflectance 37.80%	High-temperature cooling focused; not designed for low-temperature black ice-prone warning
Lima Jr. et al. (2026) [51]	Thermochromic road markings for ice and snow alerts	CIE L*a*b*, ΔE, and retroreflected luminance measurements	Road markings changed from white to pink below 0 °C; color difference ΔE reached up to 43	Road-marking system; no load-resisting mechanical evaluation

**Table 2:** Basic properties of the aliphatic polyurea resin.

Property	Value
Density (g/mL)	0.95–1.05
Viscosity (cps)	950–1400
Pot life (min)	30
Curing time (min)	20

**Table 3:** Basic properties of the reversible thermochromic pigment (R.T.P.).

Property	Value
Appearance	Red powder
Classification	Thermochromic microcapsule powder
Moisture content (%)	<2.5
Capsule wall material	Polyoxymethylene melamine
Mean particle size (μm)	5.07
Decolorization temperature range (°C)	−4.5 to 1.5
Coloration temperature range (°C)	−2.5 to 9.0

**Table 4:** Basic properties of the silica sand.

Property	Value
Particle size (mm)	1.25
Apparent density (g/cm^3^)	2.64
Unit weight (kg/m^3^)	1648
Fineness modulus	3.09
Moisture content (%)	<0.1

**Table 5:** Mix proportions of temperature-responsive polyurea-based polymer composites.

Specimen No.	Binder Content (wt.%)	Resin: Hardener	R.T.P. Dosage (phr)	Silica Sand Content (wt.%)
1	8	5:5	10	92
2	10	5:5	10	90
3	12	5:5	10	88
4	14	5:5	10	86
5	8	5:5	20	92
6	10	5:5	20	90
7	12	5:5	20	88
8	14	5:5	20	86

**Table 6:** Colorimetric properties and color difference of thermochromic polyurea-based composites at low temperature.

No.	Binder Content (wt.%)	R.T.P. (phr)	CIE L*a*b* (D65, 10°)			CIE 1931 x, y		ΔE
			L*	a*	b*	x	y	
Control	-	0	58.36	3.85	13.32	0.3567	0.3630	-
1	8	10	50.39	18.90	12.36	0.3886	0.3452	17.06
2	10	10	47.18	18.47	10.01	0.3903	0.3381	19.32
3	12	10	44.72	18.04	11.91	0.3986	0.3442	21.15
4	14	10	43.57	18.68	12.21	0.4025	0.3439	22.75
5	8	20	49.24	20.77	9.35	0.3928	0.3375	20.07
6	10	20	47.82	22.95	8.52	0.3975	0.3326	22.12
7	12	20	43.12	19.57	10.27	0.4056	0.3383	22.88
8	14	20	39.68	20.00	9.32	0.4074	0.3344	28.17

**Table 7:** Mechanical properties of thermochromic polyurea-based composite specimens.

Specimen No.	Binder Content (wt.%)	R.T.P. (phr)	Compressive Load (N)	Compressive Strength (MPa)	Flexural Load (N)	Flexural Strength (MPa)
1	8	10	12,868.3 ± 193.4	8.04 ± 0.12	1818.75 ± 2.50	4.26 ± 0.01
2	10	10	13,449.8 ± 89.9	8.41 ± 0.06	2093.75 ± 2.50	4.91 ± 0.01
3	12	10	14,297.4 ± 31.7	8.94 ± 0.02	2231.25 ± 2.50	5.23 ± 0.01
4	14	10	15,904.8 ± 69.8	9.94 ± 0.04	2543.75 ± 2.50	5.96 ± 0.01
5	8	20	14,560.2 ± 114.3	9.10 ± 0.07	2312.50 ± 2.50	5.42 ± 0.01
6	10	20	14,992.9 ± 39.7	9.37 ± 0.02	2643.75 ± 2.50	6.20 ± 0.01
7	12	20	16,146.6 ± 474.2	10.09 ± 0.30	2600.00 ± 2.50	6.09 ± 0.01
8	14	20	16,209.4 ± 405.5	10.13 ± 0.26	2268.75 ± 2.50	5.32 ± 0.01

**Table 8:** Overall quantitative evaluation of thermochromic polyurea-based composite specimens.

Evaluation Criterion	Quantitative Indicator	Most Favorable Specimen
Low-temperature color response	Highest $a^{*}$: 22.95	Specimen 6
Color contrast	Highest ${∆E}_{ab}^{*}$: 28.17	Specimen 8
Compressive strength	Highest average compressive strength: 10.13 ± 0.26 MPa	Specimen 8
Flexural strength	Highest average flexural strength: 6.20 ± 0.01 MPa	Specimen 6

## Data Availability

The original contributions presented in this study are included in the article. Further inquiries can be directed to the corresponding authors.
